# AN ECOLOGICAL MOMENTARY OUTLOOK ON SUBJECTIVE AGING

**DOI:** 10.1093/geroni/igz038.198

**Published:** 2019-11-08

**Authors:** Dikla Segel-Karpas, Amit Shrira, Margie E Lachman

**Affiliations:** 1 University of Haifa, Haifa, Israel, Israel; 2 Bar Ilan University, Ramat Gan, Israel, Israel; 3 Brandeis University, Waltham, Massachusetts, United States

## Abstract

Cross-sectional and longitudinal studies showed that subjective age – individuals’ perceptions of their own age as older or younger in relation to their actual age, is an important predictor of physical, cognitive, and mental health. Despite some initial findings suggesting that subjective aging responses to variations in the daily experiences, less is known about how daily and momentary experiences shape how old people feel, and how their perceived age affects their daily experiences. In this symposium, five studies using daily diaries and experience sampling methods will be presented and discussed to explore how subjective aging affects, and is affected by, daily changes. In the first presentation, Neupert will discuss her findings regarding the covariation of anticipatory next-day health-related stressors and coping with felt-age, suggesting that forecasting and coping with future stressors play a role in subjective aging. Presenting findings from his experience-moment-sampling study, Hughes will discuss the momentary association between subjective age and mind wandering. Zhang and Segel-Karpas will present findings from studies focusing on attitudes towards aging. Zhang will focus on state vs. trait subjective aging, exploring their association with daily variability in control and competence, while Segel-Karpas will focus on the moderating role of attitudes in the daily associations between subjective age and mental health. Finally, Shrira will present a study of rehabilitation patients, finding that subjective aging is related to physical and mental health, especially for patients with high age-awareness. Professor Lachman will lead a discussion.

